# Medical Schools and Students

**Published:** 1861-01

**Authors:** 


MEDICAL SCHOOLS AND STUDENTS.—The number of registered students in London, this year, is 1237, being an increase over last year of 132. There is also said to be a corresponding increase in the Provincial, Irish, and Scotch institutions. The London *Lancet* accounts for this difference by the wholesome educational regulations which are to be enforced in 1861, the British students thus taking advantage of the old regime. The number of students in Paris is also considerably larger than last year. In this country, on the contrary, nearly all the schools have diminished classes. In Philadelphia, the number is about 1050, as an offset against 1275 of last year.

